# Spinal Cord Stimulator Implantation for Complex Regional Pain Syndrome in Patient With Extensive Vertebral Surgical History: A Case Report

**DOI:** 10.7759/cureus.68901

**Published:** 2024-09-07

**Authors:** Munfarid A Zaidi, Mily Talati, Krishna Shah

**Affiliations:** 1 Anesthesiology, Baylor College of Medicine, Houston, USA; 2 Psychological and Brain Sciences, Washington University in St. Louis, St. Louis, USA; 3 Anesthesiology and Interventional Pain, Baylor College of Medicine, Houston, USA

**Keywords:** chronic pain management, chronic post surgical pain, complex regional pain syndrome, implanted hardware, pedicle fracture, s: percutaneous spinal cord stimulation, spinal cord stimulator

## Abstract

Spinal cord stimulation (SCS) has emerged as a novel therapeutic option for refractory complex regional pain syndrome (CRPS). However, SCS placement is often complicated by a prior history of surgical manipulation and hardware implantation along the spinal column. Through this case exploration, we aim to expand the technical approach to SCS implantation in CRPS and encourage further research into innovative approaches for this treatment modality. Our patient is a 61-year-old female with a past medical history of bilateral C7 cervical pedicle fracture status and extensive surgical manipulation, including cervical laminectomy and hardware placement along the cervical spine. The development of CRPS refractory to conventional therapies complicated her course. We obtained non-contrast computed tomography (CT) to confirm intact lamina in vertebral levels below C3 and proceeded with the SCS trial with successful lead placement up to C5. Despite prior surgical manipulation of the vertebral spine hindering our ability to access the ideal C2 level, we were able to achieve significant coverage up to the C5 level. Obtaining non-contrast CT preoperatively and carefully assessing the epidural space patency were integral to our ability to assess the feasibility of lead placement in a patient with extensive hardware. Through this approach, we are able to offer SCS to patients who would otherwise be precluded from this modality.

## Introduction

Chronic regional pain syndrome (CRPS), formerly known as reflex sympathetic dystrophy, is a complex pain disorder characterized by disproportionate pain, regional oedema, alterations in skin temperature, and motor dysfunction arising after minor injuries or trauma [1]. CRPS often profoundly impairs daily functioning [1]. The multifaceted pathophysiology involves intricate interactions between the central and peripheral nervous systems, inflammatory processes, and maladaptive neuroplasticity, complicating effective management [1,2].

The elusive aetiology of CRPS, attributed to various inciting factors such as trauma or surgery, contributes to diagnostic challenges and underscores the complexity of treatment [1,3]. Conventional therapeutic modalities include non-steroidal anti-inflammatory agents, gabapentin, physical therapy, and invasive procedures, yet a substantial proportion of patients prove refractory to these conventional modalities [1].

In recent years, spinal cord stimulation (SCS) has emerged as a novel therapeutic option for refractory CRPS. SCS, a neuromodulation technique involving the implantation of electrodes in the epidural space along the spinal cord, delivers electrical impulses that modulate pain signals, interrupt aberrant pain pathways, and align with the gate control theory of pain. However, SCS placement is often complicated by a prior history of surgical manipulation and hardware implantation along the spinal column [4,5]. This case report aims to contribute our experience and technique with such a patient. We delineate the patient's history, surgical complexities, and the successful implementation of the SCS trial. Through this case exploration, we aim to expand upon the technical approach to SCS implantation in CRPS and encourage further research into innovative approaches for this treatment modality.

## Case presentation

Our patient is a 61-year-old female who presented to Baylor St. Luke’s Medical Center for Interventional Pain with neck pain. Written informed consent was obtained from the patient to present her case.

She has a history of unprovoked fractures of the C7 bilateral cervical pedicles. Her past surgical history includes stage 1: removal of anterior fixation, anterior cervical discectomy, and fusion of C7-T1; stage 2: C2-T2 fixation and fusion, C2-3 laminectomies; and open reduction and internal fixation of C7 fracture on February 2, 2021, for loosening of hardware in the cervical spine, dysphagia, C7 bipedicular fracture, and adjacent segment disease. She later underwent removal and replacement of segmental fixation C2-T2 and drainage of the postoperative cervical fluid collection with complex muscle flap closure by plastic surgery.

She has had subsequent development of CRPS with decreased range of motion, altered sensation, skin turgor change, decreased temperature, and swelling of the right upper extremity. She developed severe pain in the midline trapezius with radiation to the occiput. She also endorsed tingling and numbness of the right fourth and fifth fingers. She tried trigger point injection, cervical medial branch block, epidural steroid injections, Tylenol-3, and Tramadol without long-term relief.

**Figure 1:**
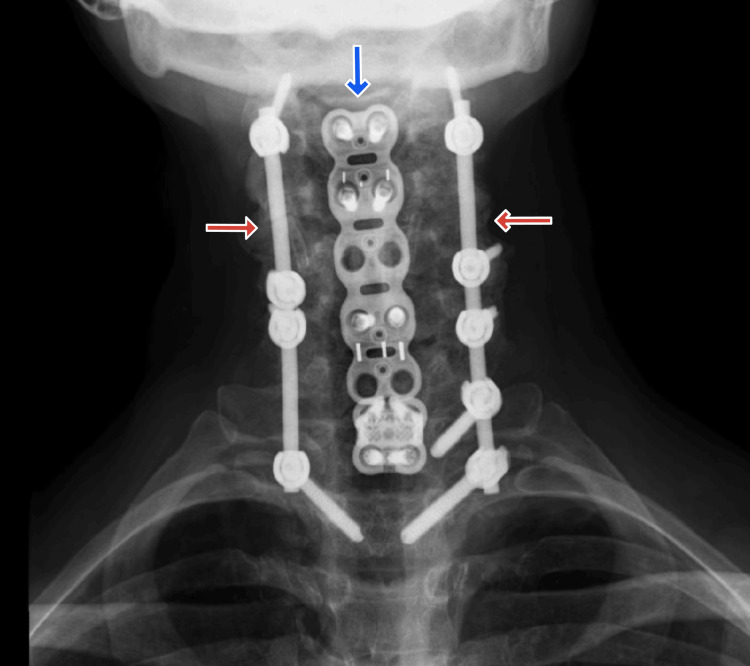
Posterior-anterior radiograph of cervical spine Posterior fixation with bilateral pedicle screws and segmental spine plating can be seen from C2-T2 levels, marked by red arrows. Anterior cervical discectomy and fusion with plate extends from C3-T1, marked by the blue arrow. Obtaining this image allows the interventional pain physician to assess the feasibility of lead placement in the goal distribution.

**Figure 2:**
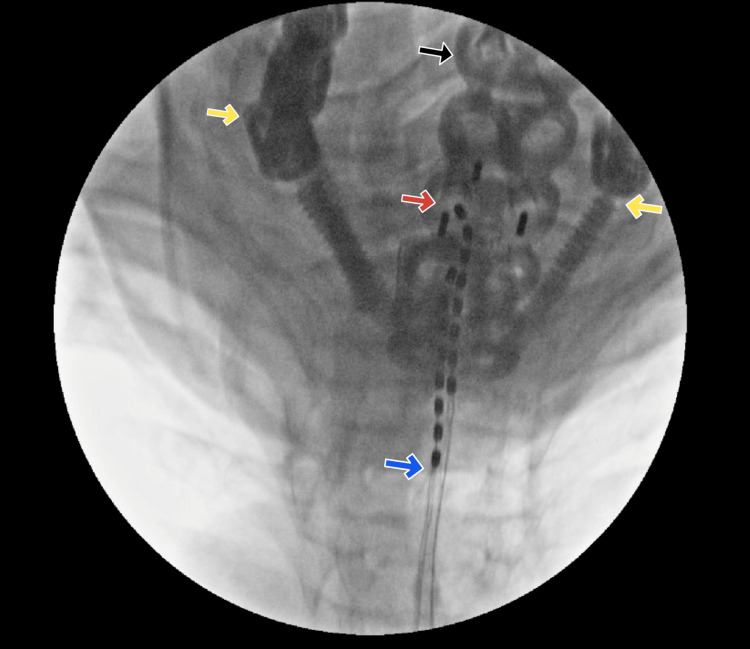
Fluoroscopic radiograph status-post SCS implantation Posterior-anterior fluoroscopic radiograph taken at the completion of SCS implantation. The blue arrow marks the inferior end of the SCS electrode; the red arrow marks the superior end (left at T1, right at C7 superiorly). The inferior ends of posterior fusion screws are indicated with yellow arrows bilaterally. The black arrow points to the plate from anterior cervical discectomy and fusion. This image highlights the successful implantation of SCS leads in a region of the cervical spine with significant overlying surgical hardware.

**Figure 3:**
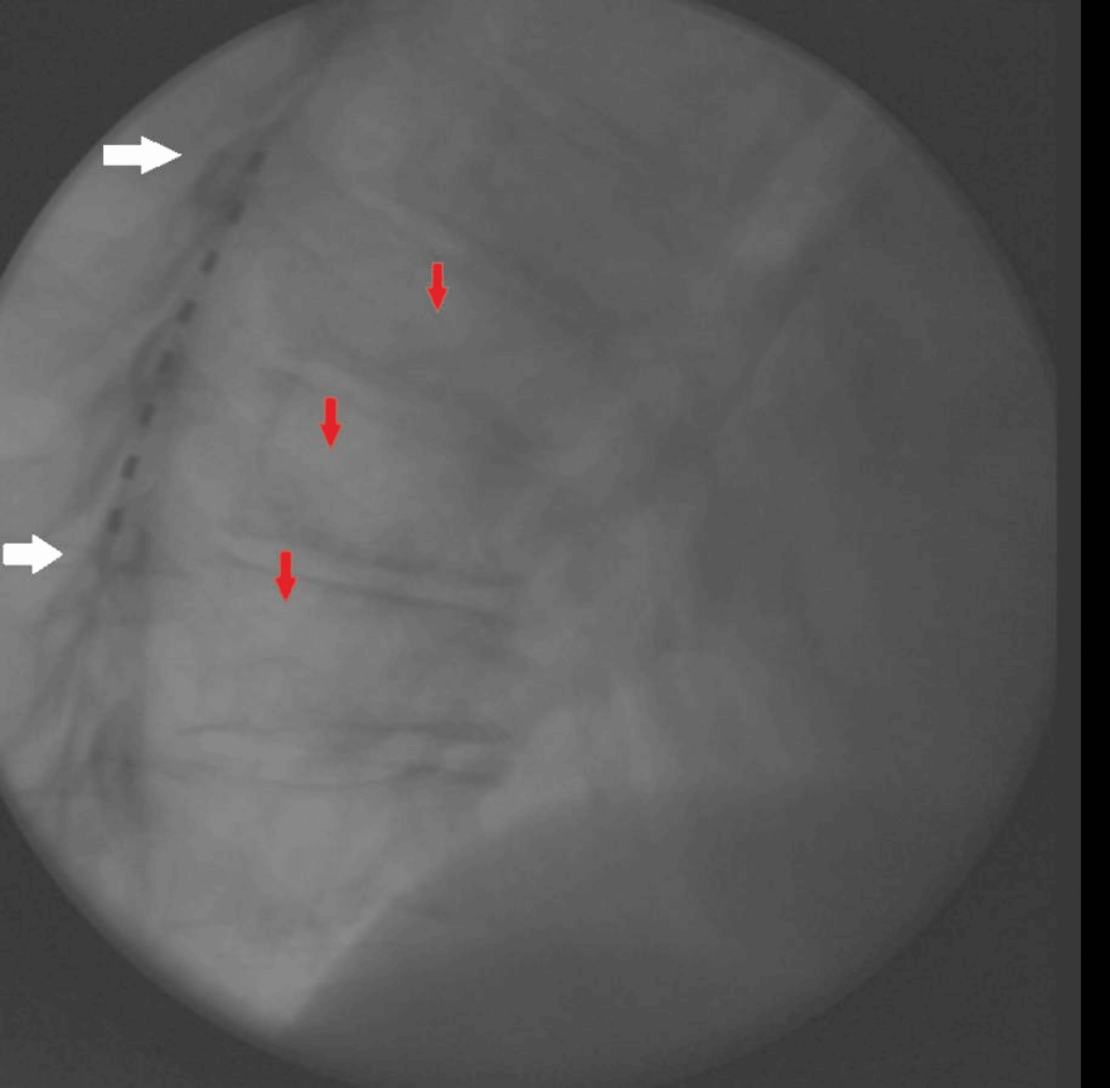
Lateral cervical fluoroscopic radiograph, post-implantation White arrows indicate the superior (at C7) and inferior edge of the SCS leads within the epidural space. Red arrows indicate posterior fusion screws. This image highlights the successful implantation of the SCS leads despite the presence of overlying surgical hardware.

Prior to our intervention, we ordered a non-contrast computed tomography (CT) scan and a radiograph to assess our patient’s anatomical variations, including lamina, bone, and epidural space (Figure 1). Via imaging, we confirmed that she is post-anterior cervical discectomy and fusion, post-posterior fusion T2-C2, and post-laminectomy C2-C3 (laminectomy obscured in radiograph). We proceeded with a SCS trial in which lead placement was achieved up to C5, and insertion was T11/12. Our patient had a >50% reduction in neck and right arm pain, as well as increased activity, function, and mood for the duration of the trial. Accordingly, we proceeded with SCS implantation (Figures 2-3).

Implantation technique

Percutaneous and surgical spinal cord stimulator placement is conducted in two stages: trial followed by final implantation. Both stages are outpatient procedures performed under fluoroscopic guidance and with standard aseptic techniques, including sterile draping.

The SCS trial, also known as a temporary or percutaneous trial, serves as a pivotal step in assessing the patient's response to electrical stimulation, gauging the extent of pain relief, and determining whether the individual is a suitable candidate for the long-term benefits of SCS [6]. Temporary leads are placed percutaneously and connected via wire to an external generator, and proper lead placement is verified by the presence of paresthesias in the appropriate dermatomal distribution [7]. If the SCS trial proves successful, permanent implantation is performed weeks later once insertion sites have healed. Radiographic images showing optimal lead placement for the trial are used by the proceduralist to guide final lead placement.

The ideal location for the SCS lead position depends on the location of pain, as referenced in Table 1. For upper extremity pain, including CRPS, leads are often placed in the upper-thoracic to lower-cervical epidural space. For low back and lower extremity pain, placement is in the lower-thoracic and upper-lumbar spaces [8,9].

**Table 1:** Spinal levels associated with anatomic locations of pain The dermatomal distribution of pain allows the interventional pain physician to plan the spinal level of their SCS lead [8-10].

Anatomic location	Spinal level
Posterior occipital region	C2
Upper extremity	C2–C5
Hand	C5–6
Chest wall angina	T1–T4
Low back	T9–T10
Thigh and knee	T9–T10
Foot	T11–L1

Programming

Paresthesia-free stimulation programs have been created where minimal to no paresthesias are felt. SCS devices contain four programmable parameters: contact selection, amplitude, pulse width, and frequency. The chosen amplitude value of the programming should provide enough stimulation to sufficiently mask the patient’s pain while avoiding discomfort. Amplitude is the measure of power output from the IPG and can be measured in current or voltage. The amplitude determines how robustly the stimulation will be felt, with higher amplitudes resulting in stronger and more intense paresthesias for tonic stimulation. We employed low-amplitude waves to achieve perceptible pain relief while minimizing uncomfortable paresthesia [7]. Pulse width, a parameter that directly correlates with amplitude, is the amount of time an electrical signal delivers power to the system, measured in microseconds. The pulse width determines how focused or broad stimulation will be felt. A higher pulse width allows for a wider area of anatomical coverage. A benefit to this approach includes a greater area of pain relief, resulting in greater neuronal recruitment. A drawback would be that the higher pulse width could potentially lead to paresthesia coverage in unwanted body distributions [7]. However, by employing low-amplitude waves, we also minimize the uncomfortable paresthesias the patients experience. Rather than more traditional tonic stimulation, we have opted to utilize burst stimulation, which is characterized by a period of high-frequency pulses of increasing amplitude followed by a brief quiescent period [7].

Our approach

Our surgical approach was similar to the standard approach, with an important exception regarding the placement of leads. As discussed above, lead placement for neck and arm pain is classically at the C2-C5 spinal level. Unfortunately, our patient’s history of C2-C3 laminectomy indicated that lead placement above the C4-5 level would subject her to an increased risk of dural puncture. The patient had also undergone significant orthopedic surgical manipulation ranging from C2-T2 with extensive hardware placement throughout these levels (Figure 1). However, given her intact lamina at all levels except C2-C3, we proceeded with a successful trial up to C5, followed by implantation (Figure 2).

Rather than more traditional tonic stimulation, we opted to utilize burst stimulation, characterized by a period of high-frequency pulses of increasing amplitude followed by a brief quiescent period [7]. Our approach is informed by recent studies, including the prospective randomized control trial of tonic versus burst SCS (SUNBURST), which demonstrated the superiority of burst stimulation for chronic trunk and/or extremity pain [11].

Clinicians performing SCS are often discouraged from attempting lead placement at spinal levels with extensive hardware due to obstruction of surgical exposure and disruption of the normal epidural anatomy upon which the lead placement technique is based. However, it is imperative to note that the epidural space has not been disrupted despite hardware placement if the lamina at those spinal levels is intact. Placement of SCS leads at these levels should, therefore, be unhindered.

## Discussion

A complex surgical history with extensive vertebral hardware and disruption of normal anatomy often poses significant challenges in the placement of SCS. In this case report, we explored the clinical presentation, history of treatment, and successful placement of SCS in a patient with a history of complex orthopedic surgical vertebral hardware, highlighting its feasibility as a therapeutic intervention.

The decision to pursue SCS in this case was guided by the patient's refractory pain despite conventional therapies and the potential benefits of neuromodulation in CRPS. As previously discussed in our report, SCS offers a targeted approach to pain management, modulating pain signals at the spinal cord level and interrupting aberrant pain pathways. With that said, the core clinical challenge of our case surrounded the obstruction of our goal dermatomes C2-C5 by prior C2-C3 laminectomy, posterior fusion C2-T2, and anterior cervical discectomy and fusion T2-C2. The successful outcome observed in our case aligns with existing literature supporting the efficacy of SCS in CRPS. Previous studies have reported significant pain relief, improved function, and enhanced quality of life with SCS, particularly in patients resistant to conventional treatments [12,13]. To the best of our knowledge, there are no case reports that specifically discuss the technical challenges of SCS implantation in CRPS patients due to obstruction of approach secondary to surgical hardware.

While our case report demonstrates the feasibility of SCS implementation in this context, several considerations warrant further exploration. Analgesic outcomes were not fully addressed in this study due to a lack of long-term pain data and warrant their own study. Challenges such as lead migration, device malfunction, and suboptimal patient selection remain pertinent considerations in SCS implementation. Lastly, comparative studies evaluating the efficacy of SCS against other interventions and the cost-effectiveness of SCS in the management of CRPS are warranted.

## Conclusions

In summary, SCS placement is often complicated by a past vertebral surgical history, with hardware placement obstructing the SCS approach. By obtaining cervical radiographs and non-contrast CT preoperatively, we can assess epidural space patency and the feasibility of lead placement in patients with extensive hardware. Through this approach, we have been able to offer SCS to patients who would otherwise have been precluded from this treatment modality. Our experience suggests that further study into the technical approach of SCS implantation in complex surgical patients is warranted.
